# Fe-Ce/Layered Double Hydroxide Heterostructures and Their Derived Oxides: Electrochemical Characterization and Light-Driven Catalysis for the Degradation of Phenol from Water

**DOI:** 10.3390/nano13060981

**Published:** 2023-03-08

**Authors:** Mihaela Mureseanu, Nicoleta Cioatera, Gabriela Carja

**Affiliations:** 1 Department of Chemistry, Faculty of Sciences, University of Craiova, Calea Bucuresti, 107I, 200478 Craiova, Romania; mihaela.mureseanu@edu.ucv.ro; 2 Faculty of Chemical Engineering and Environmental Protection, Technical University of Iasi, 71 D. Mangeron, 700050 Iasi, Romania

**Keywords:** layered double hydroxides, heterojunction, electrochemical testing, photocatalysis, phenol degradation

## Abstract

Fe-Ce/layered double hydroxides (LDHs) were synthesized via a facile route by exploiting the “structural memory” of the LDH when the calcined MgAlLDH and ZnAlLDH were reconstructed in the aqueous solutions of FeSO_4_/Ce(SO_4_)_2_. XRD analysis shows the formation of heterostructured catalysts that entangle the structural characteristics of the LDHs with those of Fe_2_O_3_ and CeO_2_. Furthermore, X-ray photoelectron spectroscopy (XPS), Raman spectroscopy, TG/DTG, SEM/EDX and TEM results reveal a complex morphology defined by the large nano/microplates of the reconstructed LDHs that are tightly covered with nanoparticles of Fe_2_O_3_ and CeO_2_. Calcination at 850 °C promoted the formation of highly crystallized mixed oxides of Fe_2_O_3_/CeO_2_/ZnO and spinels. The photo-electrochemical behavior of Fe-Ce/LDHs and their derived oxides was studied in a three-electrode photo-electrochemical cell, using linear sweep voltammetry (LSV), Mott–Schottky (M-S) analysis and photo-electrochemical impedance spectroscopy (PEIS) measurements, in dark or under illumination. When tested as novel catalysts for the degradation of phenol from aqueous solutions, the light-driven catalytic heterojunctions of Fe-Ce/LDH and their derived oxides reveal their capabilities to efficiently remove phenol from water, under both UV and solar irradiation.

## 1. Introduction

Environmental pollution, climate change and the lack of a sustainable energy supply are key challenges in the rapid development of human society [1]. The quest to provide clean water is crucial for creating a healthy life, though large quantities of pollutants, such as phenols, dyes and halobenzene compounds, are released from industry and greatly contribute to a reduction in water quality and water scarcity [2]. Due to their complex and stable chemical structure, organic pollutants cannot be removed by common treatment methods such as adsorption, extraction or biodegradation [3]. That is why there is an urgent need to find more effective degradation methods for a clean and sustainable water supply.

The photocatalytic technique promises to address the above issues, being a green and energy-saving technology which makes efficient use of solar energy, in various fields including the photocatalytic degradation of organic pollutants or hydrogen production [4]. The degradation of organic pollutants to harmless products involves the participation of active radicals such as hydroxyl (HO·) and superoxide (·O_2_^−^) produced during the reaction of H_2_O or O_2_ with photogenerated charges [5]. In order to improve photocatalytic properties, the selection of an optimal catalyst is a key step. Semiconductors are the preferred photocatalysts to target pollutant degradation due to their non-toxic nature, low cost, high stability, relatively good band edge and band gap energy positions [6]. Photocatalyst efficiency is correlated with the redox potential and relative positions of band edges of the substrate and semiconductor photocatalysts [7]. The rapid recombination of photogenerated carriers on the semiconductor surface together with low photocatalytic and low visible light harvesting efficiencies are the major disadvantages of the most studied semiconductors used as photocatalysts and limit their large-scale applications [8]. In order to overcome these impediments and to improve photocatalytic activity by modulating the energy band positions, charge transfer mechanisms and light absorption capabilities of semiconductors, some of their modification strategies developed in recent years include the construction of heterojunction systems, defect engineering or co-catalyst loading [7]. Doping of high bandgap semiconductors with non-metallic or metallic elements also provided a good response when exposed to visible light [9].

In this view, there is a lot of focus on the development of heterostuctures based on the close junction of a semiconductor unit with the LDH-type matrix, able to join the characteristics of both components. Very recent results revealed that heterostructures of the LDHs with semiconductor units, such as TiO_2_, α-Fe_2_O_3_, ZnO, Ag_2_CO_3_, CdS or g-C_3_N_4_, allow the improvement of structural properties by providing wider light absorption ranges, the inhibition of the recombination of photogenerated carriers, and consequently higher photoactivity [6]. 

Layered double hydroxides (LDHs) are two-dimensional lamellar hydroxides, presenting a similar structure to the naturally mineral hydrotalcite [10]. Their general chemical formula is: [M^2+^_1−x_M^3+^_x_·(OH)_2_]^x+^ (A^n−^_x/n_)·yH_2_O, where M^2+^ is a divalent cation (e.g., Mg^2+^, Zn^2+^, Ni^2+^, Cu^2+^); M^3+^ is a trivalent cation (e.g., Fe^3+^, Al^3+^, Cr^3+^); A^n−^ is a charge compensation anion (e.g., CO_3_^2−^, NO_3_^−^, Cl^−^), located in the interlamellar space between two brucite-like layers; and x is the stoichiometric coefficient, defined as the ratio between M^3+^/(M^2+^ + M^3+^) [11]. The physical and chemical properties of the LDHs are unique, due to their large surface area (ranging from a few m^2^/g to 100 m^2^/g), which allows their use in adsorption processes, pharmaceutical applications (due to a lack of toxicity, chemical inertia, compatibility, viability) and purification techniques [2]. LDHs have the ability to incorporate and disperse more than one transition metal cation, thus being able to regulate the redox properties of the system; therefore, they can be designed to reach specific band gap values for the hydrogen and oxygen evolution reactions [12,13] and visible light absorption [14], as well as low potentials for redox reactions [15]. LDH-based photocatalysts have demonstrated the excellent photodegradation of phenol-based compounds under visible light irradiation [16,17]. The as-synthesized MgAl/ZnAl-based LDHs have demonstrated low degradation efficiency for phenols with highly stable aromatic rings. However, their photocatalytic performances can be significantly enhanced by integrating them into heterostructures. From calcination treatments at moderate temperatures, LDHs are transformed in poorly crystalized mixtures of mixed oxides (MMOs) [18,19]. However, the structure of the most LDHs can be recovered by the ”the structural memory effect” when the derived mixed oxides are introduced in aqueous solutions containing anions [20,21]. For example, Mantilla and al. reported the mixed oxides obtained via the calcination of Zn/(Al + Fe)/LDHs as photocatalysts with a high activity towards the photodegradation of phenol and p-cresol [22]. The MMOs derived from Zn-LDHs calcinated at 300–600 °C contain additional phases of ZnO, have higher crystallinity, and a specific surface area that facilitates the charge carrier transfer and improves photocatalytic performances [23]. Recently, ZnFe-MMOs or ZnTi-MMOs have been synthesized via the calcination of their ZnFe-LDH and ZnTi-LDH precursors at different temperatures and used with very good performances in water purification processes under simulated solar light irradiation [24,25,26]. Among metal oxide-semiconductors, ceria (CeO_2_) is one of the most recognized photocatalysts due to its high oxygen storage capacity, photostability and ecological properties [27]. The increased interest in this material is determined by its wide band gap (~3.2 eV), thus being considered for applications in photocatalysis [28], sensors [29], water treatment [30] and solar cells [31]. Despite these advantages, the photocatalytic properties of CeO_2_ are restricted by the rapid recombination of photo-generated electrons and holes [32]. To entangle the photocatalytic properties of the LDH to that of CeO_2_, we previously reported on CeO_2_/Mg(Zn)Al-LDH heterostructures [33]. On the other hand, hematite (αFe_2_O_3_) with small range bandgap (~2.2 eV), which can collect visible light, is a semiconductor material widely used in water splitting and as a supercapacitor electrode due to advantages such as: good chemical stability, photocorrosion resistance [33,34,35] and absorption capacity in the region of visible light [36]. Disadvantages of this material include: low mobility and the fast recombination properties of charge carriers resulting in the 2–4 nm diffusion lengths of the minority carriers [34]. Due to low electrical conductivity and hematite metastability, Fe_2_O_3_-CeO_2_ heterostructuring might be useful to prevent the recombination of electrons and photo-generated holes, resulting in a higher efficiency of photocatalytic degradation [37]. 

Based on the above information, we present here Fe-Ce/MgLDH, Fe-Ce/ZnLDH and the mixed metal oxides derived via calcination as novel photocatalytic systems. More precisely, the structural, surface, morphological and electrochemical properties of the Fe-Ce/LDHs and their derived mixed oxides are revealed as related to the photocatalytic performances for the degradation process of phenol under the irradiation with UV or simulated solar light. Furthermore, a degradation mechanism is proposed.

## 2. Materials and Methods

### 2.1. Photocatalyst Synthesis

For the synthesis of the photocatalysts, the precursors used without further purification were: Mg(NO_3_)_2_·6H_2_O (Sigma-Aldrich, St. Louis, MO, USA), Al(NO_3_)_3_·9H_2_O (Sigma-Aldrich), Zn(NO_3_)_2_·6H_2_O (Sigma-Aldrich), Ce(SO_4_)_2_·4H_2_O (Sigma-Aldrich), Na_2_CO_3_ (Sigma-Aldrich), NaOH (Sigma-Aldrich) and FeSO_4_·7H_2_O (Sigma-Aldrich). A simple co-precipitation method was used to synthesize the LDH materials (MgAlLDH, with an Mg/Al atomic ratio = 2:1, and ZnAlLDH, with an Zn/Al atomic ratio = 3:1) as previously described [38,39]. The insertion of iron and cerium cations into the lamellar structures was achieved by the structural reconstruction of LDHs in the aqueous solution of Ce(SO_4_)_2_·4H_2_O and FeSO_4_·7H_2_O. The as-obtained solids were washed and dried in an oven at 80 °C over night. They were noted as Fe-Ce/MgAlLDH and Fe-Ce/ZnAlLDH, respectively. The corresponding MMOs were obtained after a supplementary calcination stage at 850 °C for 4 h (samples denoted Fe-Ce/MgAlLDH_850 and Fe-Ce/ZnAlLDH_850, respectively).

### 2.2. Characterization

#### 2.2.1. Material Characterization

The X-ray powder diffraction (XRD) patterns were obtained on a Rigaku Smart Lab diffractometer in the 2θ range of 10–800 with a 0.020 step, using Cu Kα radiation and a Ni filter. Phase identification was performed using the ICSD database. Raman spectra were recorded at room temperature using an in Via confocal Raman microscope (Renishaw) with a diode DPSS visible laser source (532 nm) and a Peltier cooled CCD detector. The single beam power of the laser was 25 mW and the 50× objective of the microscope was used. A Thermo Scientific (Waltham, MA, USA) (Evolution 600) spectrometer equipped with an integrated sphere was used to record the UV-Vis diffuse reflectance spectra (DRS) in the wavelength range of 200–800 nm. The morphology of the samples was evidenced using scanning electron microscopy (SEM) (SU8010 from Hitachi, Chiyoda City, Tokyo) and transmission electron microscopy (TEM) (JEOL 1200 EX II). The chemical composition was determined using an energy-dispersive X-ray spectrometer (EDX, Oxford Instruments, Abingdon, UK) coupled to SEM. The surface composition and oxidation states of the elements in the samples were investigated using X-ray photoelectron spectroscopy (XPS). XPS spectra were obtained using a Perkin-Elmer model 5500-MT spectrophotometer equipped with MgKα radiation (1253.6 eV), at 15 kV and 20 mA. TG/DTG thermogravimetric analysis was carried out using the Perkin- Elmer Pyris Diamond TG/DTA thermobalance, by heating the samples up to 850 °C, with a heating rate of 10 °C/min, in an N2 atmosphere. 

#### 2.2.2. Electrochemical Measurements

Electrochemical characterization of the samples was performed using a Zahner IM6eX electrochemical station and a potentiostat/galvanostat PG581 in a three-electrode photoelectrochemical set-up with: a working electrode with the photocatalysts spread out on a glass plate covered with fluorinated tin oxide (FTO), a Pt wire as a counter electrode, a Ag/AgCl electrode as the reference electrode, and a 2.0 M Na_2_SO_4_ solution with a pH value of 6.5 as a support electrolyte for the experiments. The working electrodes were prepared using a previously described procedure [33].

Photo-electrochemical impedance spectroscopy (PEIS) measurements were performed in the frequency range of 2.5 MHz–100 mHz with an AC amplitude of 10 mV. Mott–Schottky plots were recorded under dark conditions at a frequency of 1 kHz in the potential range −0.6 V to +0.6 V (vs. Ag/AgCl) with an amplitude of 10 mV.

#### 2.2.3. Photocatalytic Experiments

The photocatalytic activity of the investigated photocatalysts was evaluated by measuring the degradation of a 1.0 g/L phenol aqueous solution. This was performed both under irradiation with UV light and simulated solar light, after an initial stage of solution equilibration by stirring for 30 min in the dark. The suspension was stirred at a constant rate during the experiment, and the temperature was kept constant at 25 °C. Samples were collected at different irradiation times, with the overall duration of each experiment being 4 h. A 125 W UV lamp with a primary emission at a wavelength of 355 nm was used in the experiments under UV light. Tests under solar irradiation were conducted with a ScienceTech Inc. (London, ON, Canada) SLB300B (300 W xenon lamp) simulator. The Surveyor Thermo Electron HPLC system (Thermo Scientific) was used to identify, quantify and measure the degradation products as previously presented [33]. A portable spectrophotometer DR 890 with a DRB 200 thermostat and HACH LANGE cuvette testing system was used for the determination of the total organic carbon (TOC) content of the irradiated samples. The phenol photodegradation efficiency was evaluated from the conversion values that were calculated with Equation (1), where *C*_0_ and *C* are the values of initial concentration and the concentration at time *t*, respectively.

(1) $$X=\frac{C_{0}-C}{C_{0}}*100$$

## 3. Results and Discussion

### 3.1. Structural and Optical Characterization of the Photocatalysts

**Figure 1:**
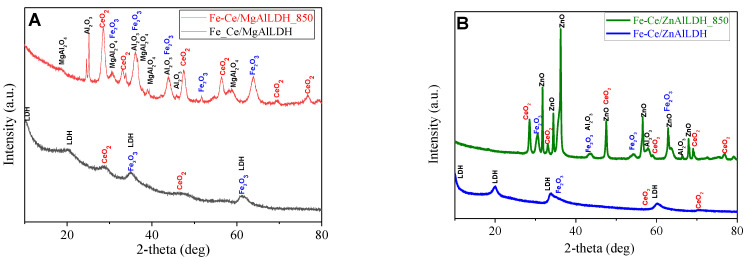
X-ray diffraction patterns of the synthesized samples: Fe-Ce/MgAlLDH (**A**) and Fe-Ce/ZnAlLDH (**B**).

The structural features of the catalysts were examined using XRD analysis (Figure 1A). The XRD pattern of Fe-Ce/MgAlLDH clearly reveals the presence of 003 and 006 diffraction peaks located at 2θ = 10.320 and 2θ = 20.350, pointing out the structural reconstruction of the LDHs with a c value of 2.57. Furthermore, the peaks from 2θ = 35.600 and 61.600 could be indexed to the 104 and 300 reflection planes of the hexagonal α-Fe_2_O_3_ (JCPDS: 33-0664), while peaks from 2θ = 28.920 and 2θ = 48.120 could be attributed to the 111 and 220 planes of the CeO_2_ fluorite structure. The EDX analysis given in Table 1 shows a Mg/Al ratio around 2 and a Zn/Al atomic ratio of ~3, while the Fe/Ce molar ratio is ~5, pointing out differences between the catalysts, which can be a consequence of the insertion of cerium cations into the LDH layers during reconstruction. After calcination, the layered structure of the original LDH was completely destroyed and additional reflections corresponding to Fe_2_O_3_, CeO_2_, Al_2_O_3_ and MgAl_2_O_4_ spinel phases can be identified in Figure 1A. 

Additionally, the distribution of CeO_2_ onto the surface of Fe_2_O_3_ via interfacial Ce-O-Fe bonding, as previously reported [40], could be considered. The characteristic peaks of the crystalized CeO_2_ phase are weak and broad (Figure 1A), indicating the presence of nanosized crystallites [41]. The amorphous character of MMOs that was already reported and is most likely related to amorphous Al^3+^ phases can generate defects which affect, in particular, the optical properties and the corresponding photocatalytic activity of these materials [42].

Figure 1B shows the XRD pattern of the Fe-Ce/ZnAlLDH where the characteristic diffraction peaks of the LDH were clearly identified and defined by the cell parameters *a* = 0.31 nm and *c* = 2.54 nm. The slight increase in the c parameter (related to the interlayer distance) may be due to the presence of the SO_4_^2−^ anions together with the intercalated charge-compensation anion CO_3_^2−^, or to the thicker metal hydroxide layers due to the addition of CeO_2_ and Fe_2_O_3_ on the surface of the brucite-like layer. The *a* parameter is consistent with that of a ZnAlLDH sample and confirms that the oxide phases of Fe_2_O_3_ with the 104 diffraction peak at 2θ = 35.250 and CeO_2_ with the 311 reflection plane (2θ = 56.120) were formed on the LDH surface. After the calcination at 850 °C, the XRD pattern (Figure 1B) presented the ZnO and Al_2_O_3_ oxide phases as the main component from the basic LDH structure and the reflections that correspond to the Fe_2_O_3_ hexagonal phase (104, 113, 018 and 214 planes) and CeO_2_ cubic fluorite (111, 200, 220, 222, 400 and 331 reflection planes). Compared to the XRD patterns of the MMOs derived from MgAlLDH, an increase in the intensity of the peaks corresponding to the crystalline phases can be noticed. Moreover, the characteristic peaks became narrower, indicating an increase in the crystallite size. A decrease in the Fe/Ce molar ratio in MMO compared to parent LDH was also noticed (5.33 compared with 5.42 for LDH sample). 

**Figure 2:**
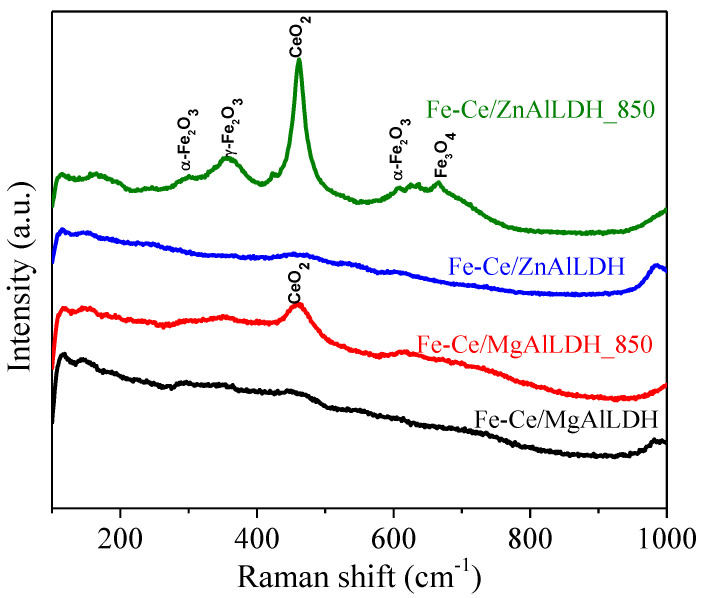
Raman spectra of as-synthesized Fe-Ce/MgAlLDH and Fe-Ce/ZnAlLDH and their calcined counterparts.

The Raman spectra of the investigated samples are shown in Figure 2. While Fe-Ce/MgAlLDH and Fe-Ce/ZnAlLDH samples have no significant bands in the investigated range, the samples calcined at 850 °C exhibited a band at about 460 cm^−1^, attributed to the fluorite phase of CeO_2_, in agreement with the XRD data. Other bands that could be ascribed to hematite (α-Fe_2_O_3_), maghemite (γ-Fe_2_O_3_) or magnetite (Fe_3_O_4_) were also evidenced in the Raman spectra of MMOs (Figure 2) [43].

**Figure 3:**
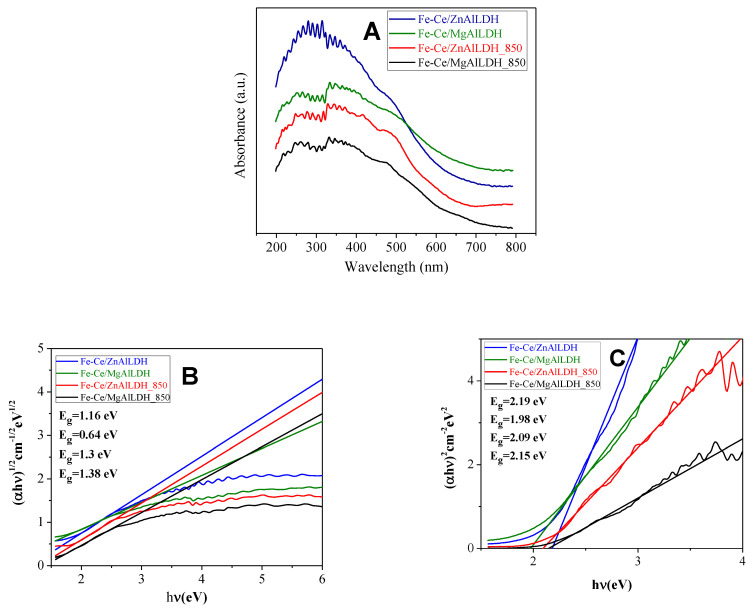
(**A**) UV-Vis diffuse reflectance spectra and plots of (**B**) (αhν)^1/2^ vs. hν and (**C**) (αhν)^2^ vs. hν for the synthesized samples: Fe-Ce/MgAlLDH, Fe-Ce/ZnAlLDH.

The UV-Vis spectroscopy was used for optical property investigations (Figure 3A). For the Fe-Ce LDH, there are two strong absorption bands at 280 nm and 340 nm, respectively, with a shoulder at 450 nm and the absorption edge extension to 650 nm, showing broad absorption in the visible-light region. This absorption profile is characteristic of the Fe_2_O_3_ present on the LDH surface, and could be attributed to the d-d transition of Fe^3+^ ions [44]. Furthermore, a broad absorption in the visible region (λ = 400–550 nm) could be the result of the photosensitizing effect of Ce^3+^ [26]. For the MMOs derived from Fe-Ce LDH, the UV-Vis spectra show the same red-shift in the optical absorption band toward the visible region that may be ascribed to a tuned transfer of light-induced electrons and holes among the semiconductor oxides of the heterojunction and the formation of some localized band gap states caused by the vacancies of Ce^3+^ [45]. The band gap energy (E_g_) was determined from the absorption spectra using the equation:

(αhν)^1/n^ = A(hν − E_g_) (2)

where α, ν and A are the absorption coefficient, light frequency and proportionality constant, as previously described [46]. For semiconductors, the band gap can be the result of a direct electron transition (n = 2) or indirect transition (n = 1/2). Figure 3B, C shows the plots of (αhν)^1/2^ vs. hν and (αhν)^2^ vs. hν of the analyzed samples. The point where the steepest slope of each graph intersects with (αhν)^1/2^ = 0 or (αhν)^2^ = 0, represents the value for E_g_. For Fe-Ce/MgAlLDH, Fe-Ce/ZnAlLDH, Fe-Ce/MgAlLDH_850 and Fe-Ce/ZnAlLDH_850 samples, the E_g_ values for n = 1/2 were 0.64, 1.16, 1.38 and 1.3 eV, and for n = 2, were 1.98, 2.19, 2.15 and 2.09 eV, respectively.

The E_g_ values of the samples when n = 2 are more credible as they are closer to the E_g_ value of 2.2 eV for Fe_2_O_3_ [37], confirming a direct electron transition. Fe_2_O_3_, as a narrow band gap semiconductor, could be a possible donor of electrons and a sensitizer under visible light irradiation for a wide band gap semiconductor such as ZnO and CeO_2_. In the solid solution of mixed oxides, there is a new band structure with orbitals from metals and oxygen combined in a structure with a lower band gap than those of semiconductor oxides components. Comparing the values of the band gap of the Fe-Ce/MgAlLDH and Fe-Ce/ZnAlLDH samples, a difference of 0.21 eV is observed, which indicates that the photocatalyst composition has a significant influence on the E_g_ value of the semiconductor material [47]. Band gap energies for uncalcined Fe-Ce/MgAlLDH and Fe-Ce/ZnAlLDH samples were 1.98 eV and 2.19 eV, respectively. After calcination, there was a slight increase in the band gap for Fe-Ce/MgAlLDH_850 to 2.15 eV and a slight decrease for Fe-Ce/ZnAlLDH_850 to 2.09 eV. These effects are due to different interactions between the CeO_2_ and Fe_2_O_3_ semiconductor oxides. Furthermore, the Ce^3+^/Ce^4+^ ratio in the photocatalyst could influence the band gap position, knowing that a higher concentration of Ce^4+^ produces a narrower band gap that allows photocatalytic activity under visible light. As the XPS spectra revealed, both Ce^3+^ and Ce^4+^ cationic species have been identified in the analyzed samples.

**Figure 4:**
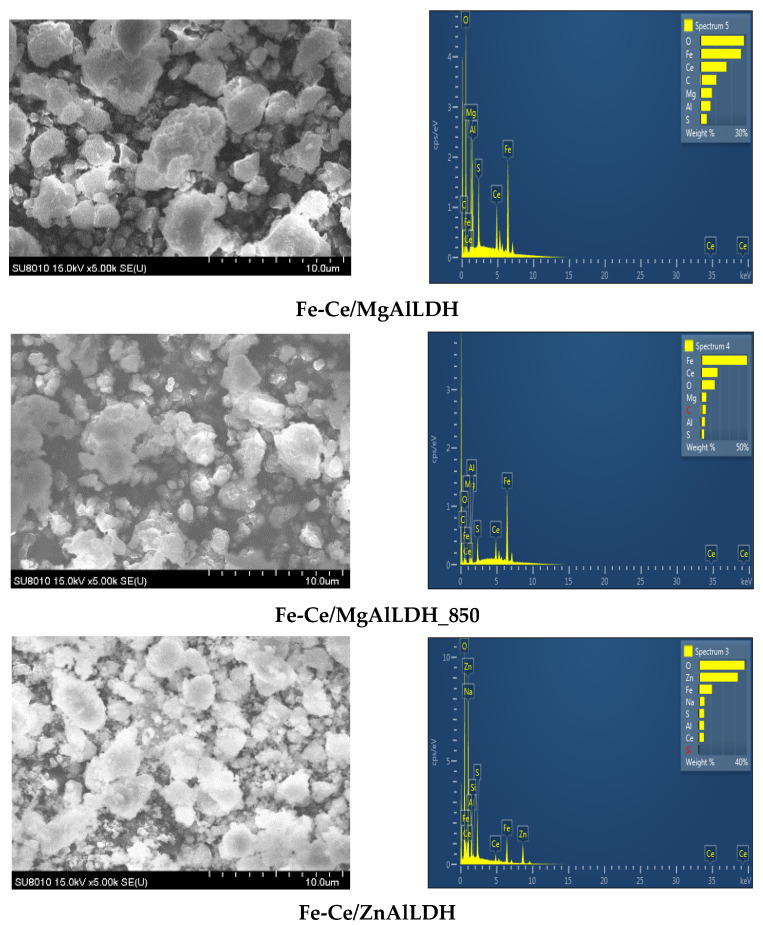

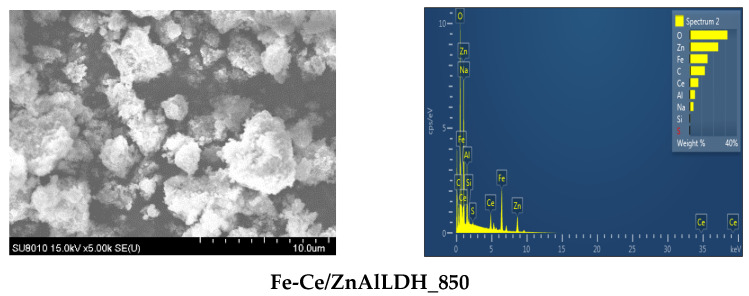
SEM images and EDX spectra of the as-synthesized Fe-Ce/MgAlLDH and Fe-Ce/ZnAlLDH and their calcined counterparts.

In Figure 4, the SEM images and the corresponding EDX spectra of the synthesized Fe-Ce/LDH samples are shown. The morphology of the Fe-Ce/MgAlLDH sample is revealed as agglomerates with a sponge-like aspect that increased the heterogeneity of the surface, with CeO_2_ and Fe_2_O_3_ nanoparticles randomly dispersed on the LDH surface. After the calcination of Fe-Ce/MgAlLDH at 850 °C, the stratified structure of the sample was destroyed and metal oxide crystallization occurred. The disappearance of the characteristic peaks for the lamellar structure in the X-ray diffractograms (Figure 1) supports the collapse of this structure during the calcination process. The Fe-Ce/ZnAlLDH photocatalyst presents the same morphology as Fe-Ce/MgAlLDH, specific to the morphology of LDHs but with much more interconnected particles, which reveals a wider distribution of their sizes and a greater surface heterogeneity due to the smaller particles of CeO_2_ and Fe_2_O_3_ dispersed on their surface. After Fe-Ce/ZnAlLDH calcination, larger and more agglomerated metal oxide particles were formed, which further increased the heterogeneity of their surface.

**Figure 5:**
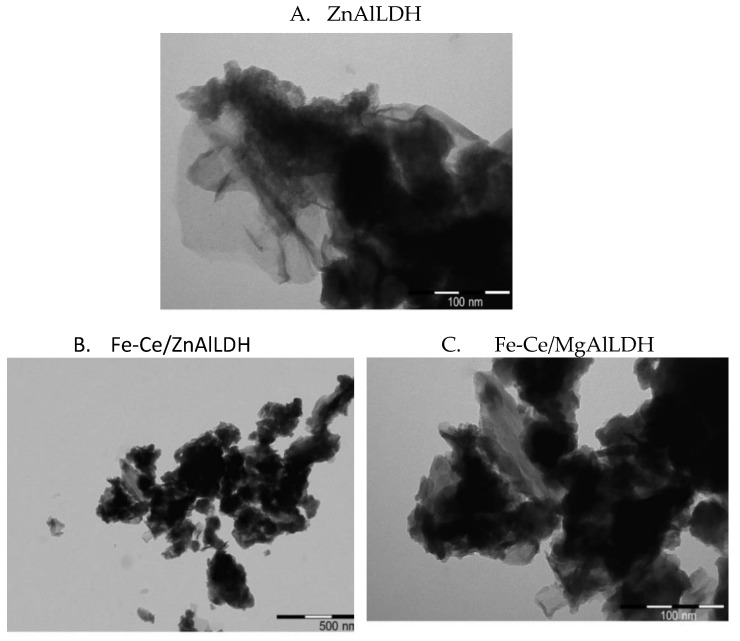

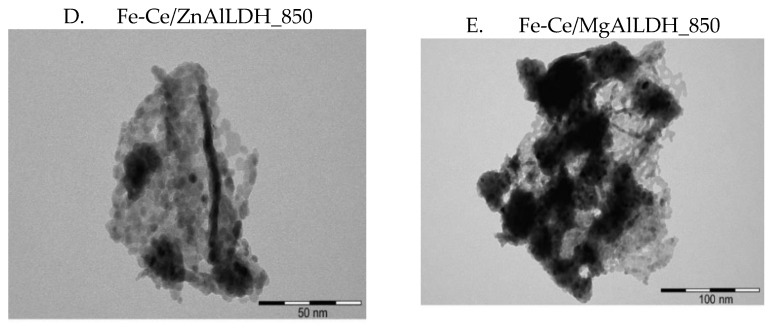
TEM images of: ZnAlLDH (**A**), Fe-Ce/ZnAlLDH (**B**), Fe-Ce/MgAlLDH (**C**), Fe-Ce/ZnAlLDH_850 (**D**), and Fe-Ce/MgAlLDH_850 (**E**).

The structural characteristic of the reconstructed Fe-Ce LDHs and the corresponding MMOs were also revealed via TEM analysis (Figure 5). As show in Figure 5A, the ZnAlDLH samples showed large interconnected platelet nanoparticles, with an average size of 200 nm, typical for LDH morphology [48]. In the reconstructed Ce-Fe/ZnAlLDH samples (Figure 5B), the nanoparticles of Fe_2_O_3_ and CeO_2_ (~25 nm) are highly dispersed on the surface of the LDH particles, almost completely covering them. For the Ce-Fe/MgAlLDH sample (Figure 5C), a morphology similar to the ZnAlLDH counterpart is observed. TEM images of the MMOs reveal the Fe_2_O_3_ and CeO_2_ nanoparticles (~30 nm) entangled with the irregular aggregates of crystalline and amorphous oxide particles, probably resulting from the collapse of the stratified structure and partial crystallization of the mixed oxides.

### 3.2. XPS Analysis

**Figure 6:**
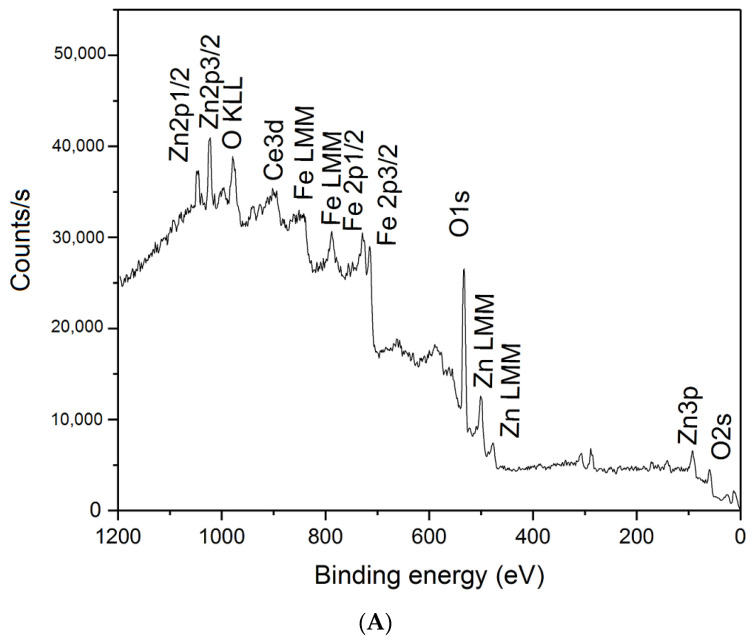

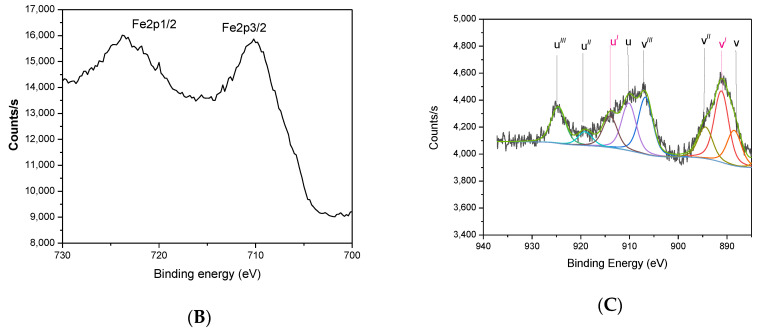
The XPS spectra of Fe-Ce/ZnAlLDH_850 sample including: (**A**) a wide survey and high resolution spectra of (**B**) Fe2p and (**C**) Ce3d, respectively.

In order to further investigate the composition and the chemical states of different species in the Fe-Ce/Mg(Zn)AlLDH_850 samples, X-ray photoelectron spectroscopy (XPS) analyses were performed. The wide survey spectrum in Figure 6 illustrated the binding energy peaks which were attributed to O1s, Fe2p, Ce3d and Zn2p. As shown in Figure 6B, the peaks located at 710.2 and 723.6 eV in the Fe2p high resolution XPS spectra are attributed to Fe2p_3/2_ and Fe2p_1/2_, respectively [45]. The energy of these peaks corresponds to Fe^3+^ from Fe_2_O_3_ [49] and confirms the presence of this iron oxide in the MMOs obtained after calcination of the Fe-Ce/ZnAlLDH sample. 

Figure 6C presents the high resolution Ce3d spectra and the deconvolution results which can be assigned to 3d_5/2_ and 3d_3/2_ spin-orbit states denoted as u and v, respectively. Both Ce^4+^ (u, u″, u‴, v, v″, and v″″) and Ce^3+^ (u′ and v′ 0) chemical states of cerium were evidenced [50]. This result confirms that a mixture of Ce^3+^/Ce^4+^ oxidation states exists on the sample surface. Usually, when Ce^3+^ is present, charge deficiency is compensated by oxygen vacancies in the lattice of CeO_2_, resulting in the formation of oxygen defects [51]. These ones are important both for the enhancement of the activity and stability of the photocatalyst and for the activation of hydrogen production from oxygen-containing bonds, such as that in the water-splitting reaction. Furthermore, they act as an electron scavenger and generate superoxide radicals. The presence of Ce^4+^ in a higher concentration could be correlated with a narrower bandgap and a better activity of ceria in the visible region of light spectra, which is the case of the analyzed MMO sample (Table 2).

### 3.3. Thermal Analysis (TGA-DTA)

The thermal stability of the LDH-type samples was determined as a function of temperature via thermogravimetric analysis coupled with differential thermal analyses (TG-DTA).

**Figure 7:**
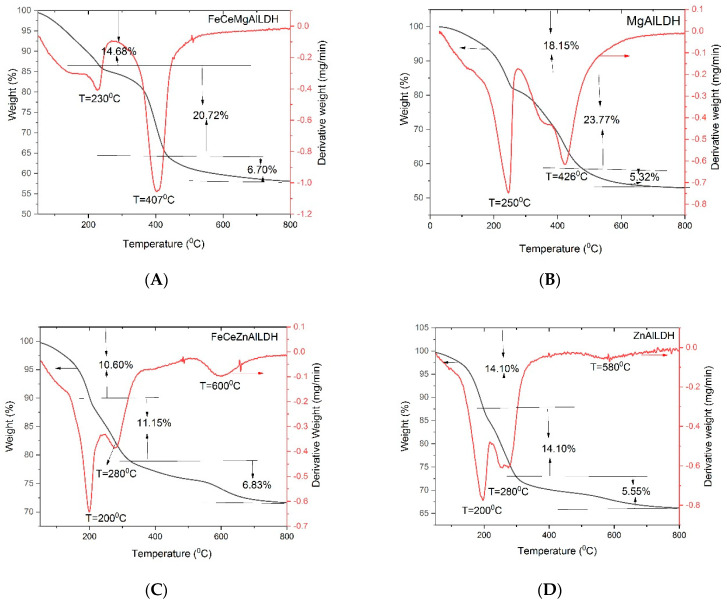
The TG/DTG profiles of (**A**) Fe-Ce/MgAlLDH, (**B**) MgAlLDH, (**C**) Fe-Ce/ZnAlLDH and (**D**) ZnAlLDH samples.

Figure 7 presents the TG/DTG profiles for the LDHs modified after reconstruction with Fe and Ce and for the pristine Mg(Zn)AlLDHs. The total mass loss for the Fe-Ce/MgAlLDH sample was 42.10%, and for the MgAlLDH precursor, it was 47.24%. For these samples, there were two endothermic peaks centered at 230 °C and 407 °C, for Fe-CeMgAlLDH and 250 °C and 426 °C for the MgAlLDH sample, respectively. The first peak can be attributed to the release of water molecules physically adsorbed, or from interlayer region, and the second one corresponds to the decomposition of carbonate and dehydroxylation of brucite-like layers. Over this temperature range, the LDH-type sample underwent dehydroxylation and decarbonation reactions and the formation of metal oxides and spinel species. For Fe-Ce/ZnAlLDH and ZnAlLDH samples, total mass loss was lower than for their counterparts based on MgAlLDH (28.93% for Fe-Ce/ZnAlLDH and 34.04% for ZnAlLDH). In addition, the temperature ranges of thermal decomposition were also different, characterized by endothermic peaks located at lower temperature values than for the samples with MgAlLDH. In this regard, the first mass loss occurred at 200 °C and the second one at 280 °C (Fe-Ce/ZnAlLDH) or 260 °C (ZnAlLDH). This behavior demonstrates the easier loss of water and decomposition of the compensation anions. A supplementary endothermic peak was located at 600 °C in the thermogram of the Fe-Ce-ZnAlLDH sample, corresponding to a greater amount of metal oxides and spinel species. The better crystallinity of these samples was sustained by their XRD profile (Figure 1).

### 3.4. Photoelectrochemical Study 

The current density voltage (I-V) curves were carried out in an aqueous solution of 0.2 M Na_2_SO_4_, in dark and light illumination conditions both for hydrotalcites layered materials (Fe-Ce/MgAlLDH, Fe-Ce/ZnAlLDH) and their corresponding mixed oxides (Fe-Ce/MgAlLDH_850, Fe-CeZnAlLDH_850) in order to evaluate their photo electrochemical properties. The results of photocurrent measurements on the prepared LDH- or MMO-type photo electrodes showed that bare MgAlLDH and ZnAlLDH and their corresponding MMOs yielded a very low current density over the entire voltage scan window and were considered for further comparison with the Ce/Fe-modified materials (Table 2). For all tested materials, the current starts to generate at around −1 V vs. Ag/AgCl (−0.42 V vs. RHE) and increases toward the anodic direction with respect to the applied bias either under dark or illumination conditions, denoting their n-type semiconductor properties. A slight shift toward more negative potentials can be seen for the samples under illumination (Table 2), indicating a more favorable oxidation reaction on the surface. A lower band bending for the photo-generated charge carrier separation and an accelerated transport of the charges at the electrode–electrolyte interface can explain this behavior [52]. The photocurrent density was greater under illumination for all electrodes, confirming the semiconductor properties of the tested electrode materials. The increase in the current density for the Fe-Ce-modified LDH-type samples indicates a narrower band gap for these new materials, which allowed an enhanced absorption of visible light (Table 2, Figure 4). Furthermore, there was a synergetic effect of Fe_2_O_3_ and CeO_2_ highly dispersed on the MgAlLDH or ZnAlLDH surface that facilitated electron transport. For the MMOs photocatalysts, a vectorial transfer of the photogenerated electrons and holes among Fe_2_O_3_-CeO_2_-ZnO mixed oxides could be considered, hindering their recombination [45]. Analyzing the current density at an onset potential of 0.5 V vs. Ag/AgCl (Table 2), the best results were obtained for that Fe-Ce/ZnAlLDH_850 sample that showed an increase with one order of magnitude for the current density when switching from dark to simulated solar illumination. Moreover, the corresponding LDH precursor sample exhibited the highest value for the current density under investigation conditions. These results can be correlated with the smallest Mott–Schottky slope and the highest photoelectron life time.

**Table 2:** Photo-electrochemical properties of LDH- and MMO-type electrodes.

Electrode	Voc (V) vs. Ag/AgCl		Current Density (µA/cm^2^) at 0.5 V vs. Ag/AgCl		V_fb_ (V) vs. Ag/AgCl ^1^	Band Gap E_g_ (eV) ^2^	E_VB_^3^
	Dark	Illum.	Dark	Illum.			
Fe-Ce/MgAlLDH	−0.13	−0.18	1.11	6.52	−0.40 (0.18)	1.98	2.16
Fe-Ce/MgAlLDH_850	−0.21	−0.23	1.37	2.68	−0.48 (0.10)	2.15	2.25
Fe-Ce/ZnAlLDH	−0.13	−0.21	17.8	55.53	−0.63 (−0.05)	2.19	2.14
Fe-Ce/ZnAlLDH_850	−0.15	−0.19	0.68	6.63	−0.67 (−0.09)	2.09	2.00

^1^ From M-S plots (in parenthesis are the potential in V vs. RHE); ^2^ From DRUV spectra; ^3^ E_VB_ = E_fb_ + E_g_.

In order to investigate the electrical properties of the new synthesized photocatalysts, electrochemical impedance spectroscopy measurements were performed. The Mott–Schottky (M-S) plots of all the tested anodes allowed the evaluation of the flat band potential (V_fb_) as the intercept of the linear fit of the plot C^−2^ vs. the applied potential bias with the potential axis (Table 2). According to the M-S plots, the positive slope indicates that all the investigated photocatalysts are n-type semiconductors. For this type of semiconductor, the conduction band corresponds approximately to the flat band potential. As the slope of the Fe-Ce/ZnAlLDH sample is the smallest, this photocatalyst probably presented the highest density of charge carriers. A higher charge donor density is an important factor for electronic conductivity within the photoelectrode, which can also improve the efficiency of photogenerated electron-hole pair separation and transport. Generally, after the calcination of the Ce/Fe LDH samples, a decrease in carrier density can be noticed. Even if for the calcined samples the carrier density was smaller, the shift of Vfb toward more negative values can compensate for this decrease. 

**Figure 8:**
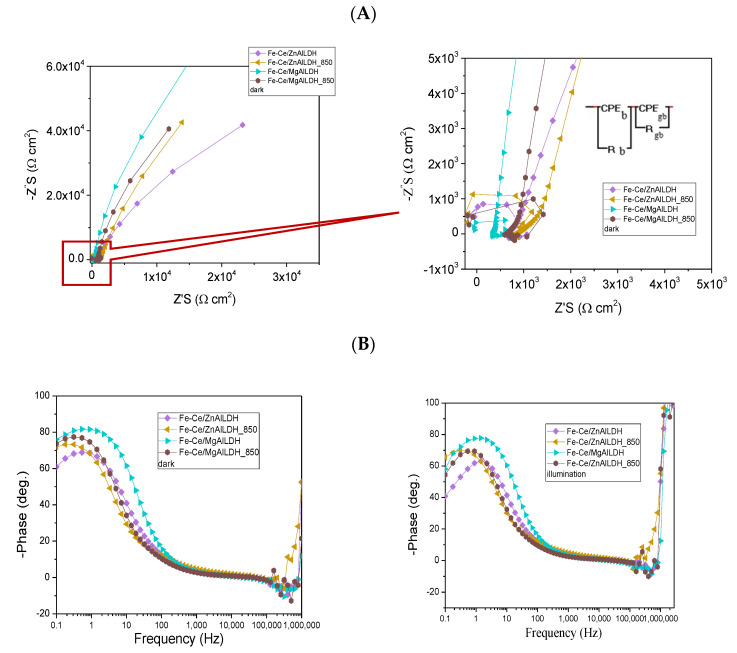
EI spectra of photo anodes in open circuit vs. Ag/AgCl reference electrode. (**A**) Nyquist plots and (**B**) Bode phase plots.

To further explore the electrochemical behavior of the electrodes based either on Fe/Ce-modified LDH or their corresponding mixed oxides obtained from their calcination at 8500C, photo electrochemical impedance spectroscopy (PEIS) measurements were performed in the frequency range 100 mHz–2.5 MHz. Nyquist plots were obtained for the LDH- or MMO-type samples in order to investigate change in charge transfer resistance under illumination (Figure 8A). An EIS Spectrum Analyser software was used to fit the Nyquist plots of the impedance spectra recorded under illumination [53]. A constant phase element (CPE) was used to simulate a non-ideal capacitor, being usually assigned to the inhomogeneity, surface roughness, porosity and tortuosity of the electrode materials. Therefore, a CPE in parallel with a resistance appears as a depressed semicircle in the Nyquist plot [54]. The equivalent circuit model used to fit the Nyquist plots of the EI spectra is shown in the inset of Figure 8A. The order of magnitude of the CPE values was further used to assign features such as grain/bulk (10^−12^ F) from a high frequency region or grain boundaries (10^−11^–10^−8^ F, depending mostly on their microstructure) in a low frequency region [55]. The sample Fe-CeZnAlLDH_850 exhibited the lowest total electrical resistance among the investigated samples (Table 3), in agreement with the previous photoelectrochemical parameters.

The Bode plots of EI spectra are shown in Figure 8B, evidencing a change in the phase angle in the 60–75° range, which could be ascribed to the partial redox and capacitive nature of Ce/Fe-modified LDH-type samples or their corresponding MMOs [33]. The Bode plots allow the evaluation of photoelectrons’ life times (*τ*), this parameter being correlated to the maximum frequency peak (*f_peak_*) from the low frequency region according to Equation (3) [56]:

(3) $$\tau =\frac{1}{\left(2\pi f_{peak}\right)}$$

The life time of the photoelectrons for all tested photocatalysts is shown in Table 4. The highest photoelectron life time was calculated for the sample Fe-Ce/ZnAlLDH, indicating a more facile electron transport at the electrode/electrolyte interface and a decreased probability for electron and hole recombinations.

**Figure 9:**
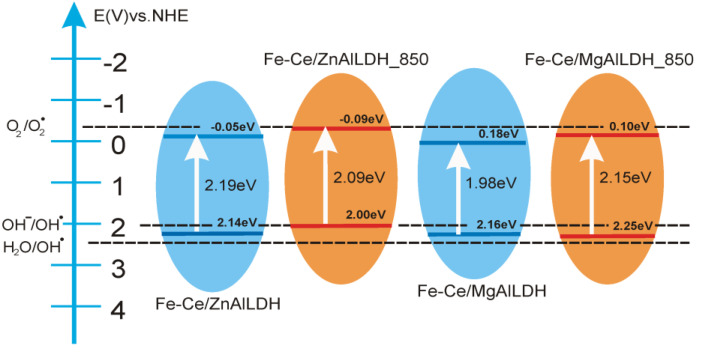
Diagrams of energy levels for the tested photocatalysts.

The diagrams of energy levels for the new heterostructured LDH and MMO photocatalysts obtained in this study were constructed using the values of band gap energy (E_g_) calculated from UV-Vis spectra, those of conduction band (E_CB_) from photoelectrochemical measurements, and valence band (E_VB_) calculated as the sum of E_g_ and E_fb_ (Figure 9). According to these diagrams, the photocatalyst materials with a suitable potential for water reduction (more negative than the hydrogen reduction potential of −0.58 V (vs. Ag/AgCl, calculated at pH 6.5) are Fe-Ce/ZnAlLDH and Fe-Ce/ZnAlLDH_850 (Table 2, Figure 9). Moreover, all the investigated photocatalysts are active in the water oxidation reaction as their oxidation potential is more positive than the oxidation potential of H_2_O (+0.65 V vs. Ag/AgCl at the same pH of 6.5). It is well known that the photocatalysts with suitable potential for both H_2_ and O_2_ evolution could be used for water splitting. Therefore, all investigated LDH- and MMO-type photocatalysts could be efficient in the advanced oxidation reactions (AOR) of different organic pollutants from aqueous media.

### 3.5. Photocatalytic Activity

**Figure 10:**
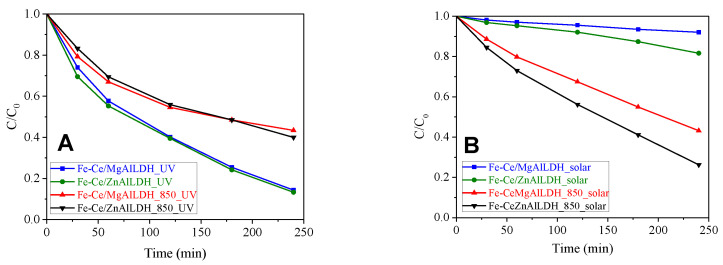
Photocatalytic degradation of phenol under (**A**) UV and (**B**) simulated solar light irradiation in the presence of different LDH-based catalysts.

The photocatalytic activity of the Fe-Ce/layered double hydroxides and their derived oxides was also investigated. The samples were tested for the photocatalytic degradation of phenol in aqueous solution, under illumination with UV and simulated solar light. In the absence of the catalyst, the phenol photolysis was less than 5%. In a previous study, the phenol photodegradation activity of the parent MgAlLDH, ZnAlLDH and their corresponding MMOs was tested in the same conditions [33]. Only the Zn-containing samples presented photocatalytic activity (20% phenol degradation for ZnAlLDH and 25% for the corresponding MMO, after 4 h irradiation with UV light), with lower degradation yields under solar light irradiation (2% and 5% for the same samples), and were not further considered for comparison. The results of phenol photodegradation tests performed on Fe-Ce-modified LDH-based materials assessed using degradation rate (*C*/*C*_0_) vs. reaction time (*t*) are shown in Figure 10. 

The best results were obtained for the samples based on ZnAlLDH. The ZnO presented as a supplementary phase either in the reconstructed Fe-Ce/ZnAlLDH, or the corresponding Fe-Ce/MMOs enhanced photocatalytic efficiency in the phenol degradation reaction both due to its intrinsic high photocatalytic activity and the synergistic effects of heterojunction with the other semiconductor metal oxides (Fe_2_O_3_ and CeO_2_). Furthermore, the Zn-containing samples presented a higher Fe/Ce atomic ratio, as the chemical composition from EDX analysis revealed (Table 1). The availability in a greater proportion of Fe_2_O_3_ could enhance the separation of photo-generated electron-hole pairs [57] and extend the visible response [58], with both effects being beneficial for photocatalytic activity. The presence of ceria in these heterostructured photocatalysts allows a superior photocatalytic activity due to the redox properties of Ce^4+^/Ce^3+^, by facilitating the formation of labile oxygen vacancies and a high mobility of the bulk oxygen species. Moreover, it allows the transfer of the electron from the 4f orbital of Ce^3+^ to absorbed oxygen with the formation of superoxide radicals and the prevention of the recombination of the photo-generated charges [27] In the photocatalytic tests under UV irradiation, the best degradation efficiency was obtained for the LDH-reconstructed catalysts, reaching a maximum phenol conversion of 87% (for Fe-Ce/ZnAlLDH). In the same time, MMOs gave better results in photocatalytic degradation under irradiation with simulated solar light (74% phenol degradation for Fe-Ce/ZnAlLDH_850 sample). It is worth mentioning that the textural and structural properties of LDH and MMO photocatalysts are quite different. For the LDH-type photocatalysts, large nano/microplates of the reconstructed LDHs are tightly covered with nanoparticles of Fe_2_O_3_ and CeO_2_. A better separation and lower recombination of charges could be a consequence of their high dispersion on the LDH support, leading to increased photoactivity. Calcination at 850 °C of the LDH precursors promoted the formation of the highly crystallized mixed oxides of Fe_2_O_3_/CeO_2_/ZnO and spinels with a homogeneous distribution of the component phases. In these type of structures, CeO_2_/Fe_2_O_3_/ZnO(MgAl_2_O_4_) heterojunctions can be built up, allowing a directed migration and separation of charge carriers; thus, the oxidation and reduction reactions occur at spatially separated active sites, preventing the recombination of charge carriers (electrons and holes). These characteristics are beneficial for the pollutant photodegradation reaction. As expected, the photocatalysts with the best phenol degradation results also presented the right energy band values that allow its advanced degradation by active radicals (·O_2_^−^ and ·OH) (Figure 9). 

The first-order Langmuir–Hinshelwood kinetic model represented by Equation (4) was used to establish the kinetic parameters and the optimal time for phenol degradation under irradiation with UV light and simulated solar light.

(4) $$\ln\left(\frac{C}{C_{0}}\right)=-K_{app}t$$

where *C*_0_ and *C*—initial and final phenol concentration, *K_app_*—apparent constant of the first order reaction rate in min^−1^, and *t*—reaction time.

All the experiments were performed for 4 h to have a common basis of comparison between them. In Table 5, both the *K_app_* values (from the graph ln (*C*/*C*_0_) vs. reaction time (*t*)) and the correlation coefficient, R^2^, are reported for all samples tested under UV and simulated solar light irradiation. All heterostructures fit well with this first-order kinetic model and consequently the value of the apparent rate constant follows the same order. For the photocatalyst with the best photocatalytic activity (Fe-Ce/ZnAlLDH), the value of the *K_app_* is nine times higher in UV than in visible light (0.0079 min^−1^ and 0.0009 min^−1^, respectively). All the experiments were performed for 4 h to have a common basis of comparison between them. 

From the comparison of phenol photodegradation activity of the new synthesized heterostrucures based on Fe-Ce/LDHs with other LDH-type photocatalysts (Table 6), we can state that the photocatalytic systems we propose show high efficiency.

**Figure 11:**
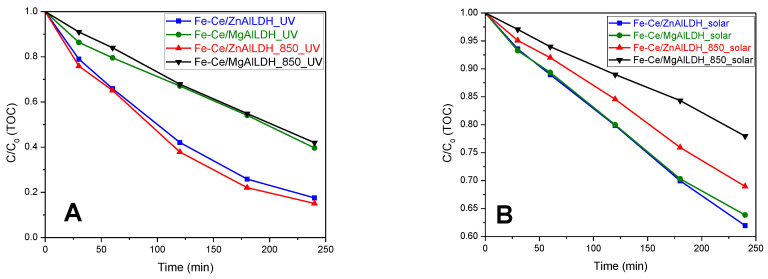
Evolution of total organic carbon (TOC) vs. reaction time under (**A**) UV and (**B**) simulated solar light irradiation.

The mineralization degree of organic pollutants was evaluated by determining the total organic carbon (TOC). Figure 11 shows the degradation of organic compounds via the phototcatalysis process by measuring the total organic carbon concentration. Under UV light, the best mineralization is obtained with the photocatalyst Fe-Ce/ZnAlLDH_850, reaching a value of 85%, while under simulated solar light, the highest mineralization was 38% for the Fe-Ce/ZnAlLDH sample. It is obvious that the photocatalysts with the best activity presented the greatest mineralization degree of the organic pollutant. As the kinetic studies revealed (Table 5), the phenol degradation reaction was slower under solar light illumination. A lower mineralization degree was expected for the same duration of the experiment than under UV conditions.

The main intermediates which resulted from the phenol degradation, identified after HPLC analysis, were maleic acid, fumaric acid, hydroquinone and resorcinol. Their presence sustains that the photodegradation process involve ·O_2_^−^ and ·OH radical species [33].

**Scheme 1:**
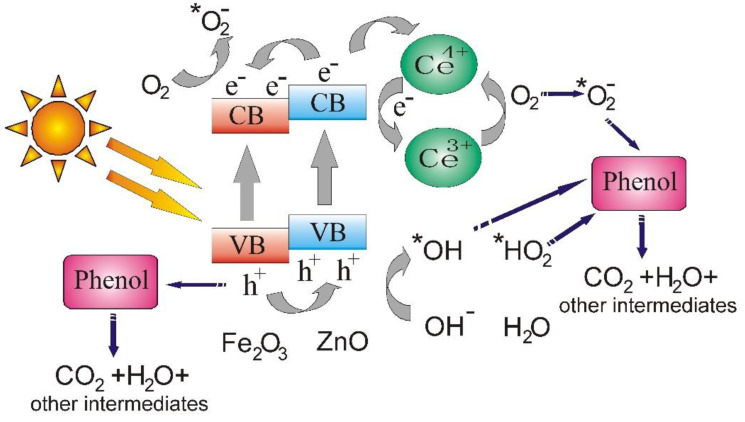
Possible mechanism of phenol photodegradation (* was used to mark the radicals).

On the basis of the above analysis and discussion, we proposed a possible mechanism of phenol photodegradation catalyzed by the Fe-Ce-ZnAlLDH_850 heterostructure (Scheme 1). Thus, after sample irradiation with UV/solar light, the electrons undergo a jump from the valence band (VB) to the conduction band (CB) of the semiconductor metal oxides of the heterojunction and left the holes in their valence band. Due to a different band position of ZnO and Fe_2_O_3_ semiconductors, after their photo activation, it is possible that electrons (e^−^) transfer from the CB of ZnO to the CB of Fe_2_O_3_ and the holes (h^+^) can transfer from the VB of Fe_2_O_3_ to the VB of ZnO. The photogenerated charges in both semiconductors can react with O_2_ to form ·O^2−^ (e^−^) and with H_2_O or HO^−^ to form ·OH (h^+^). Furthermore, electrons in the CB of Fe_2_O_3_ and ZnO can be transferred to Ce^4+^ on the surface of CeO_2_ to generate Ce^3+^ ions, which can react with O_2_ molecules to further generate ·O^2−^ and Ce^4+^. The main intermediates resulting from the phenol degradation identifiedafter HPLC analysis were maleic acid, fumaric acid, hydroquinone and resorcinol. Their presence sustains that the photodegradation process involve ·O^2−^ and ·OH radical species [33]. The photodegradation of phenol occurs in several steps as follows: (i) phenoxy radical formation with activation of para and orto positions conducting to hydroquinone and catechol intermediate; (ii) benzoquinone formation and aromatic ring opening to form aliphatic (muconic, maleic, oxalic, formic) acids; and (iii) mineralization to carbon dioxide and water. 

Beside this mechanism in which phenol degradation occurs mainly based on photogenerated radicalic species, a ligand-to-metal charge-transfer (LMCT) mechanism could also be considered, as previously reported for other LDH-type photocatalysts [66]. In this case, the adsorption of the phenol on the catalyst surface is essential and the molecules are attached to the LDH surface either via a phenolate linkage reaction or physisorbed via hydrogen bonding [61]. Under UV or simulated solar light irradiation, an electron could be transferred from the ligand (phenol) to the metal sites of the photocatalyst (most likely to the 4f band of CeO_2_) and thereafter to an electron acceptors such as O_2_, forming a radical species that attacks the pollutant molecule.

In summary, the synthesized materials can be efficiently used for phenol photodegradation under solar light by generating ·O^2−^ and HO^−^ reactive oxygen species that allow the mineralization of the pollutant down to the CO_2_ and H_2_O end-products.

## 4. Conclusions

Fe-Ce/LDHs heterostructures have been obtained using a simple and inexpensive synthesis method, consisting of the reconstruction of the LDHs in an aqueous solution of iron and cerium sulphates. The corresponding MMOs have been obtained from the calcination of Fe-Ce/LDHs at 850 °C. Their structural, textural and electrochemical characterization revealed a close junction between Fe_2_O_3_-CeO_2_ and the LDH nanounits. The reconstructed LDHs and the derived MMO revealed better photocatalytic performances in comparison to the “as synthesised” LDHs in the process of phenol degradation from aqueous solutions due to the increase in charge separation and the extended visible optical response. The activity and stability of the new materials have been improved in the presence of the Ce^4+^/Ce^3+^ couple, while their electrochemical potentials make the novel materials suitable for hydrogen and oxygen evolution reactions.

## Figures and Tables

**Table 1:** Compositional and structural parameters for LDH-type photo catalysts.

Sample	Mg/Al Molar Ratio	Zn/Al Molar Ratio	Fe/Ce Molar Ratio	LDH Cell Parameters (nm)	
				*a*	*c*
Fe-Ce/MgAlLDH	1.99	-	5.07		2.57
Fe-Ce/MgAlLDH_850	2.32	-	5.28	-	-
Fe-Ce/ZnAlLDH	-	2.90	5.42	0.31	2.54
Fe-Ce/ZnAlLDH_850	-	3.06	5.33	-	-

**Table 3:** Electrical resistances of LDH- and MMO-type electrodes.

Sample	R_b_, Ω	R_gb_, Ω
Fe-CeZnAlLDH	347	1.24 × 10^5^
Fe-CeZnAlLDH_850	633	7.03 × 10^4^
Fe-CeMgAlLDH	702	8.09 × 10^4^
Fe-CeMgAlLDH_850	861	2.35 × 10^5^

**Table 4:** Photo-electrochemical properties of LDH- and MMO-type electrodes.

Electrode	Mott–Schottky Slope	Photoelectron Life Time (*τ*)	
		Dark	Illum.
Fe-Ce/MgAlLDH	176.61	1.11	6.52
Fe-Ce/MgAlLDH_850	193.33	1.37	2.68
Fe-Ce/ZnAlLDH	4.20	17.8	55.53
Fe-Ce/ZnAlLDH_850	87.95	0.68	6.63

**Table 5:** Kinetic parameters for the photocatalytic degradation of phenol.

Sample	UV Light Irradiation		Simulated Solar Light Irradiation	
	*K_app_* (min^−1^)	R^2^	*K_app_* (min^−1^)	R^2^
Fe-Ce/MgAlLDH	0.0077	0.9948	0.0003	0.8936
Fe-Ce/ZnAlLDH	0.0079	0.9906	0.0009	0.9457
Fe-Ce/MgAlLDH_850	0.0033	0.9288	0.0034	0.9969
Fe-Ce/ZnAlLDH_850	0.0037	0.9721	0.0054	0.9907

**Table 6:** Phenol photodegradation on different LDH photocatalysts.

Photocatalyst	Phenol Initial Conc. (mg/L)	Light Source	Time	Phenol Degrad. (%)	Ref.
NiFe-LDH	20 mL, 20 ppm	UV λ_max_ = 510 nm	120 min		[59]
Ag@Ag_3_PO_4_/g-C_3_N_4_/NiFeLDH		Sunlight	2 h	90%	[14]
NiFe-LDH/N-rGO/g-C_3_N_4_	20 mL, 20 ppm	125 W medium Hg lamp (λ_max_ = 420 nm)	120 min	75%	[12]
ZnAlLDH/Ag	20 mg/mL	300 W Xenon lamp	210 min	80%	[60]
CeO_2_/MgAlLDH	80 mg/L	UV lamp (115 V, 254 nm, 4400 μW/cm^2^, without filter)	7 h	50	[61]
ZnAl and ZnGaAlLDH and MMOs	40 mg/L 80 mg/L	UV lamp (115 V, 254 nm, 4400 μW/cm^2^, without filter)	6 h	80 60	[62]
Mn-doped ZnAlLDH	30 mg/L	UV lamp (254 nm and 2.8 W)	6 h	95	[63]
SnO_2_-ZnAlLDH	40 mg/L	UV lamp (254 nm and 4.4 mW/cm^2^)	2 h	91	[64]
ZnAlCeLDH Ce (3.5%, 5.0%, and 10% mol)	40 mg/L	UV lamp (254 nm and 4400 μW/cm^2^)	4 h	90	[26]
ZnAlLa MMOs	40 mg/L	UV-Vis lamp (254 nm, 4400 μW/cm^2^)	300 min	97	[65]
CeO_2_/ZnSn-LDH	50 mg/L	UV lamp	4 h	92	[66]
Fe-Ce/ZnAlLDH Fe-Ce-MMOs	20 mg/L 20 mg/L	UV lamp (125 W) Xenon lamp (300 W)	4 h 4 h	87 74	This work

## Data Availability

The data presented in this study are available on request from the corresponding author.

## References

[1] Gielen D., Boshell F., Saygin D., Bazilian M.D., Wagner N., Gorini R. (2019). The role of renewable energy in the global energy transformation. Energy Strateg. Rev..

[2] Zhang G., Zhang X., Meng Y., Pan G., Ni Z., Xia S. (2020). Layered double hydroxides-based photocatalysts and visible-light driven photodegradation of organic pollutants. A review. Chem. Eng. J..

[3] Mukherjee S., Roychaudhuri U., Kundu P.P. (2018). Bio-degradation of polyethylene viacomplete solubilisation by the action of pseudomonas fluorescens bio-surfactant produced by bacillus licheniformis and anionic surfactant. J. Chem. Technol. Biotechnol..

[4] Montoya J.H., Seitz L.C., Chakthranont P., Vojvodic A., Jaramillo T.F., Norskov J.K. (2017). Materials for solar fuels and chemicals. Nat. Mater..

[5] Goktas S., Goktas A. (2021). A comparative study on recent progress in efficient ZnO based nanocomposite and heterojunction photocatalysts: A review. J. Alloys Compd..

[6] Prasad C., Tang H., Liu Q.Q., Zulfiqar S., Shah S., Bahadur I. (2019). An overview of semiconductors/layered double hydroxides composites: Properties, synthesis, photocatalytic ana photoelectrochemical applications. J. Mol. Liq..

[7] Wang H., Li X., Zhao X., Li C., Song X., Zhang P., Huo P., Li X. (2022). A review on heterogeneous photocatalysis for environmental remediation: From semiconductors to modification strategies. Chin. J. Catal..

[8] Cui X., Tian L., Xian X., Tang H., Yang X. (2018). Solar photocatalytic water oxidation over Ag_3_PO_4_/g-C_3_N_4_ composite materials mediated by metallic Ag and graphene. Appl. Surf. Sci..

[9] Wang S., Yun J.-H., Luo B., Butburee T., Peerakiatkhajohn P., Thaweesak S., Xiao M., Wang L. (2017). Recent Progress on Visible Light Responsive Heterojunctions for Photocatalytic Applications. J. Mater. Sci. Technol..

[10] Forno C., Hibino T., Leroux F., Taviot-Gueho C. (2006). Layered double hydroxides. Appl. Clay Sci..

[11] Kang D., Yu X., Tong S., Ge M., Zuo J., Cao C., Song W. (2013). Performance and mechanism of Mg/Fe layered double hydroxidea for fluoride and arsenate removal from aqueous solution. Chem. Eng. J..

[12] Nayak S., Parida K. (2019). Deciphering Z-scheme charge transfer dynamics in heterostructure NiFe-LDH/NrGO/g-C_3_N_4_ nanocomposite for photocatalytic pollutant removal and water splitting reactions. Sci. Rep..

[13] Nayak S., Swain G., Parida K. (2019). Enhanced Photocatalytic Activities of RhB Degradation and H_2_ Evolution from in Situ Formation of the Electrostatic Heterostructure MoS2/NiFe LDH Nanocomposite through the Z-Scheme Mechanism via p-n Heterojunctions. ACS Appl. Mater. Interf..

[14] Nayak S., Parida K. (2018). Dynamics of charge-transfer behavior in a plasmon-induced quasi-type-II p-n/n-n dual heterojunction in Ag@Ag_3_PO_4_/g-C_3_N_4_/NiFe LDH nanocomposites for photocatalytic Cr(VI) reduction and phenol oxidation. ASC Omega.

[15] Chen S., Thind S.S., Chen A. (2016). Nanostructured materials for water splitting—State of the art and future needs: A mini-review. Electrochem. Commun..

[16] Ao Y., Wang D., Wang P., Wang C., Hou J., Qian J. (2016). Enhanced photocatalytic properties of the 3D flower-like Mg-Al layered double hydroxides decorated with Ag_2_CO_3_, under visible light illumination. Mater. Res. Bull..

[17] Zhao P., Liu X., Tian W., Yan D., Sun X., Lei X. (2015). Adsolubilization of 2,4,6-trichlorophenol from aqueous solution by surfactant intercalated ZnAl layered double hydroxides. Chem. Eng. J..

[18] Balsamo N., Mendieta S., Oliva M., Eimer G., Crivello M. (2012). Synthesis and Characterization of Metal Mixed Oxides from Layered Double Hydroxides. Proc. Mater. Sci..

[19] Zhu J.Y., Zhu Z.L., Zhang H., Lu H.T., Qiu Y.L., Zhu L.Y., Küppers S. (2016). Enhanced photocatalytic activity of Ce-doped Zn-Al multi-metal oxide composites derived from layered double hydroxide precursors. J. Colloid Interf. Sci..

[20] Carja G., Grosu E.F., Mureseanu M., Lutic D. (2017). A family of solar light responsive photocatalysts obtained using Zn^2+^Me^3+^ (Me=AlGa) LDHs doped with Ga_2_O_3_ and In_2_O_3_ and their derived mixed oxides: A case study of phenol/4-nitrophenol decomposition. Catal. Sci. Technol..

[21] Zhang L., Liu J., Xiao H., Liu D., Qin Y., Wua H., Li H., Dub N., Hou W. (2014). Preparation and properties of mixed metal oxides based layered double hydroxide as anode materials for dye-sensitized solar cell. Chem. Eng. J..

[22] Mantilla A., Tzompantzi F., Fernández J.L., Góngora J.A.I.D., Gómez R. (2009). Photodegradation of phenol and cresol in aqueous medium by using Zn/Al+Fe mixed oxides obtained from layered double hydroxides materials. Catal. Today.

[23] Lee S.-B., Ko E.-H., Park J.Y., Oh J.-M. (2021). Mixed metal oxides by calcination of Layered Double hydroxide: Parameters affecting specific surface area. Nanomaterials.

[24] Di G., Zhu Z., Zhang H., Zhu J., Lu H., Zhang W., Qiu Y., Zhu L., Kueppers S. (2017). Simultaneous removal of several pharmaceuticals and arsenic on Zn-Fe mixed metal oxides: Combination of photocatalysis and adsorption. Chem. Eng. J..

[25] Chuaicham C., Karthikeyan S., Song J.T., Ishihara T., Ohtani B., Sasaki K. (2020). Importance of ZnTiO_3_ phase in ZnTi-mixed metal oxide photocatalysts derived from layered double hydroxide. ACS Appl. Mater. Interf..

[26] Suarez-Quezada M., Romero-Ortiz G., Suarez V., Morales-Mendoza G., Lartundo- Rojas L., Navarro Ceron E., Tzompantzi F., Robles S., Gomez R., Mantilla A. (2016). Photodegradation of phenol using reconstructed Ce doped Zn/Al layered double hydroxides as photocatalysts. Catal. Today.

[27] Ma R., Zhang S., Wena T., Gu P., Li L., Zhao G., Niu F., Huang Q., Tang Z., Wang X. (2019). A critical review on visible-light-response CeO_2_-based photocatalysts with enhanced photooxidation of organic pollutants. Catal. Today.

[28] Furle P., Scheffe J.R., Steinfeld A. (2012). Syngas production by simultaneous splitting of H_2_O and CO_2_
*via* ceria redox reactions in a high-temperature solar reactor. Energy Environ. Sci..

[29] Izu N., Shin W., Murayama N., Kanzaki S. (2002). Resistive Oxygen Gas Sensors Based on CeO_2_ Fine Powder Prepared Using Mist Pyrolysis. Sens. Actuat. B.

[30] Lu X.H., Zheng D.Z., Gan J.Y., Liu Z.Q., Liang C.L., Liu P., Tong Y.X. (2010). Porous CeO_2_ nanowires/nanowire arrays: Electrochemical synthesis and application in water treatment. J. Mater. Chem..

[31] Corma A., Atienzar P., Garcia H., Ching J.Y.C. (2004). Hierarchically mesostructured doped CeO_2_ with potential for solar-cell use. Nat. Mater..

[32] Mao L., Wang Y., Zhong Y., Ning J., Hu Y. (2013). Microwave-assisted deposition of metal sulfide/oxide nanocrystals onto a 3D hierarchical flower-like TiO_2_ nanostructure with improved photocatalytic activity. J. Mater. Chem. A.

[33] Chivu V., Gilea D., Cioatera N., Carja G., Mureseanu M. (2020). Heterostructures of Ce-Ti/layered double hydroxides and the derived MMOs for photoenergy applications. Appl. Surf. Sci..

[34] Wu H., Wu L., Shen S., Liu Y., Cai G., Wang X., Qiu Y., Zhong H., Xing Z., Tang J. (2020). Enhanced photoelectrochemical performance of an α-Fe_2_O_3_ nanorodsphotoanode with embedded nanocavities formed by helium ions implantation. Int. J. Hydrogen Energy.

[35] Ma H., Hwang J.B., Chae W.S., Chung H.S., Choi S.H., Mahadik M.A., Lee H.H., Jang J.S. (2021). Magnetron sputtering strategy for Zr-Fe_2_O_3_ nanorod photoanode fabricated from ZrOx/β-FeOOH nanorods for photoelectrochemical water splitting. Appl. Surf. Sci..

[36] Luan P., Xie M., Liu D., Fu X., Jing L. (2014). Effective charge separation in the rutile TiO_2_ nanorod-coupled α-Fe_2_O_3_ with exceptionally high visible activities. Sci. Rep..

[37] Sabari Arul N., Mangalaraj D., Ramachandran R., Nirmala Grace A., In Han J. (2015). Fabrication of CeO_2_/Fe_2_O_3_ composite nanospindles for enhanced visible light driven photocatalyst and supercapacitor electrode. J. Mater. Chem. A.

[38] Mureseanu M., Georgescu I., Bibire L.E., Carja G. (2014). Cu^II^(Sal-Ala)/MgAlLDH and Cu^II^(Sal-Phen)/MgAlLDH as novel catalytic systems for cyclohexene oxidation by H_2_O_2_. Catal. Commun..

[39] Mikami G., Grosu F., Kawamura S., Yoshida Y., Carja G., Izumi Y. (2016). Harnessing self-supported Au nanoparticles on layered double hydroxides comprising Zn and Al for enhanced phenol decomposition under solar light. Appl. Catal. B Environ..

[40] Machida M., Kawada T., Fujiri H., Hinokuma S. (2015). The Role of CeO_2_ as a Gateway for Oxygen Storage over CeO_2_-Grafted Fe_2_O_3_ Composite Materials. J. Phys. Chem. C.

[41] Lahkale R., Sadik R., Elhatimi W., Bouragba F.Z., Assekouri A., Chouni K., Rhalmi O., Sabbar E. (2022). Optical, electrical and dielectric properties of mixed metal oxides derived from Mg-Al Layered Double Hydroxides based solid solution series. Phys. B Phys. Cond. Matter.

[42] Perkowitz S. (1993). Optical Characterization of Semiconductors: Infrared, Raman, and Photoluminescence Spectroscopy.

[43] Slavov L., Abrashev M.V., Merodiiska T., Gelev C., Vandenberghe R.E., Markova-Deneva I., Nedkov I. (2010). Raman spectroscopy investigation of magnetite nanoparticles in ferrofluids. J. Magn. Magn. Mater..

[44] Seftel E.M., Puscasu M., Mertens M., Cool P., Carja G. (2015). Photo-responsive behavior of γ-Fe_2_O_3_ NPs embedded into ZnAlFe-LDH matrices and their catalytic efficiency in wastewater remediation. Catal. Today.

[45] Lei Y., Huo J., Liao H. (2018). Fabrication and catalytic mechanism study of CeO_2_-Fe_2_O_3_-ZnO mixed oxides on double surfaces of polyimide substrate using ion-exchange technique. Mater. Sci. Semicond. Proc..

[46] Mureseanu M., Filip M., Somacescu S., Baran A., Carja G., Parvulescu V. (2018). Ce, Ti modified MCM-48 mesoporous photocatalysts: Effect of the synthesis route on support and metal ion properties. Appl. Surf. Sci..

[47] Li J., Wang S., Sun G., Gao H., Yu X., Tang S., Zhao X., Yi Z., Wang Y., Wei Y. (2021). Facile preparation of MgAl_2_O_4_/CeO_2_/Mn_3_O_4_ heterojunction photocatalyst and enhanced photocatalytic activity. Mater. Today Chem..

[48] Gilea D., Lutic D., Carja G. (2019). Heterostructures of silver and zinc based layered double hydroxides for pollutant removal under simulated solar light. Environ. Eng. Manag..

[49] Zhou X., Liu J.Y., Wang C., Sun P., Hu X.L., Li X.W., Shimanoe K., Yamazoe N., Lu G.Y. (2015). Highly sensitive acetone gas sensor based on porous ZnFe_2_O_4_ nanospheres. Sens. Actuat. B Chem..

[50] Mureseanu M., Chivu V., Osiac M., Ciobanu M., Bucur C., Parvulescu V., Cioatera N. (2021). New photoactive mesoporous Ce-modified TiO_2_ for simultaneous wastewater treatment and electric power generation. Catal. Today.

[51] Kusmierek E. (2020). A CeO_2_ Semiconductor as a Photocatalytic and Photoelectrocatalytic Material for the Remediation of Pollutants in Industrial Wastewater: A Review. Catalysts.

[52] Chaudhary D., Singh S., Vankar V.D., Khare N. (2017). A ternary Ag/TiO_2_/CNT photoanode for efficient photoelectrochemical water splitting under visible light irradiation. Int. J. Hydrogen Energ..

[53] EIS Spectrum Analyser. http://www.abc.chemistry.bsu.by/vi/analyser/.

[54] Cioatera N., Parvulescu V., Rolle A., Vannier R.N. (2012). Enhanced ionic conductivity of Sm, Gd-doped ceria induced by modification of powder synthesis procedure. Ceram. Int..

[55] Irvine J.T.S., Sinclair D.C., West A.R. (1990). Electroceramics: Characterization by Impedance Spectroscopy. Adv. Mater..

[56] Chen K., Feng X., Hu R., Li Y., Xie K., Li Y., Gu H. (2013). Effect of Ag nanoparticle size on the photoelectrochemical properties of Ag decorated TiO_2_ nanotube arrays. J. Alloys Compd..

[57] Xia S., Meng Y., Zhou X., Xue J., Pan G., Ni Z. (2016). Ti/ZnO–Fe_2_O_3_ composite: Synthesis, characterization and application as a highly efficient photoelectrocatalyst for methanol from CO_2_ reduction. Appl. Catal. B Environ..

[58] Shooshtari N.M., Ghazi M.M. (2017). An investigation of the photocatalytic activity of nano α-Fe_2_O_3_/ZnO on the photodegradation of cefixime trihydrate. Chem. Eng. J..

[59] Nayak S., Parida K. (2021). Comparison of NiFe-LDH based heterostructure material towards photocatalytic rhodamine B and phenol degradation with water splitting reactions. Mat. Today Proc..

[60] Lestari P.R., Takei T., Yanagida S., Kumada N. (2020). Facile and controllable synthesis of Zn-Al layered double hydroxide/silver hybrid by exfoliation process and its plasmonic photocatalytic activity of phenol degradation. Mater. Chem. Phys..

[61] Valente J.S., Tzompantzi F., Prince J. (2011). Highly efficient photocatalytic elimination of phenol and chlorinated phenols by CeO_2_/MgAl layered double hydroxides. Appl. Catal. B Environ..

[62] Prince J., Tzompantzi F., Mendoza-Damian G., Hernandez-Beltran F., Valente J.S. (2015). Photocatalytic degradation of phenol by semiconducting mixed oxides derived from Zn(Ga)Al layered double hydroxides. Appl. Catal. B Environ..

[63] Morales-Mendoza G., Alvarez-Lemus M., López R., Tzompantzi F., Adhikari R., Lee S.W., Torres-Martínez L.M., Gómez R. (2016). Combination of Mn oxidation states improves the photocatalytic degradation of phenol with ZnAlLDH materials without a source of O_2_ in the reaction system. Catal. Today.

[64] Mendoza-Damián G., Tzompantzi F., Mantilla A., Pérez-Hernández R., Hernández-Gordillo A. (2016). Improved photocatalytic activity of SnO_2_–ZnAl LDH prepared by one step Sn^4+^ incorporation. Appl. Clay Sci..

[65] Tzompantzi F., Mendoza-Damian G., Rico J.L., Mantilla A. (2014). Enhanced photoactivity for the phenol mineralization on ZnAlLa mixed oxides prepared from calcined LDHs. Catal. Today.

[66] Seftel E.M., Puscasu M.C., Mertens M., Cool P., Carja G. (2015). Fabrication of CeO_2_/LDHs self-assemblies with enhanced photocatalytic performance: A case study on ZnSn-LDH matrix. Appl. Catal. B Environ..

